# The Therapeutic Strategies for SLE by Targeting Anti-dsDNA Antibodies

**DOI:** 10.1007/s12016-021-08898-7

**Published:** 2021-09-20

**Authors:** Yaqi Wang, Shengxiang Xiao, Yumin Xia, Huixia Wang

**Affiliations:** Department of Dermatology, The Second Affiliated Hospital of Xi’an Jiaotong University, Xi’an, China

**Keywords:** Anti-dsDNA antibody, Systemic lupus erythematosus (SLE), Target therapy, Therapeutic peptide, B cell

## Abstract

Systemic lupus erythematosus (SLE) is a chronic autoimmune disease characterized by diverse serological autoantibodies. Anti-dsDNA antibodies are involved in multiple organ damage, especially the kidney, skin, and central nervous system. Anti-dsDNA antibodies play a pivotal role in SLE, and researchers have developed therapeutic strategies targeting these antibodies. Approaches to reduce anti-dsDNA antibodies via B cell targeted biologics against B cell surface antigens, B cell survival factors, or Bruton’s tyrosine kinase have effectively eliminated B cells. However, their non-specific depletion hampers normal immune system functioning and limits the therapeutic benefits. Thus, scientists have attempted anti-dsDNA antibodies or lupus-specific strategies, such as the immature dendritic cell vaccine and immunoadsorption. Recently, synthetic mimic peptides (hCDR1, pCONs, DWEYS, FISLE-412, and ALW) that directly block anti-dsDNA autoantibodies have attracted attention, which could ameliorate lupus, decrease the serological autoantibody titer, reduce the deposition of renal autoantibodies, and improve pathological performance. These potent small peptide molecules are well tolerated, non-toxic, and non-immunogenic, which have demonstrated a benign safety profile and are expected to be hopeful candidates for SLE management. In this review, we clarify the role of anti-dsDNA antibodies in SLE, mainly focus on the current strategies targeting anti-dsDNA antibodies, and discuss their potential clinical value.

## Introduction

Systemic lupus erythematosus (SLE) is a chronic autoimmune disease associated with severe organ damage involving both the innate and adaptive immune systems. Although the precious etiopathogenesis of SLE is not fully defined, growing evidence suggests that the clearance deficiency of apoptotic and necrotic cells and the degradation deficiency of NETosis (neutrophil extracellular traps) may play a vital role in SLE, especially in the early stages [1, 2]. That is, the autoantigens contain nuclear substances released from the dead cells mentioned above, which are exposed to antigen-presenting cells (APCs), e.g., follicular dendritic cells and macrophages. After processing by APC, autoantigens are presented to autoreactive T cells or B cells. In this process, diverse immunological pathways dysregulation, proinflammatory cytokine secretion, autoantibodies production, and complement system activation ultimately lead to the loss of autoimmune tolerance [3]. Dozens of multiple autoantibodies can be detected in the serum of SLE patients, including anti-dsDNA, anti-Smith, anti-ribonucleoprotein (RNP), anti-Ro, anti-La, rheumatoid factor (RF), anti-nuclear (ANA), and anti-phospholipid [4]. Additionally, numerous autoantibodies are the hallmark of the SLE serologic profile.

These autoantibodies may be associated with certain clinical manifestations, especially the strong correlation between anti-dsDNA antibodies and lupus nephritis. Anti-dsDNA antibodies could directly bind to DNA antigens or cross-reactive antigens in renal cells and indirectly form anti-dsDNA antibody-containing immune complexes (ICs) in the renal parenchyma. This triggers the complement cascade, accompanied by an infiltration of immune cells and cytokine release, which can induce kidney inflammatory and fibrotic processes [5]. Anti-dsDNA IgG can penetrate living cell by binding to nuclear components to internalize autoantibodies, which can induce mesangial cell proliferation and increase the secretion of proinflammatory cytokine IL-6 [6, 7]. Although the exact penetration mechanism of anti-dsDNA IgG is still unclear, its unique penetration nature may augment it to a more prominent pathogenetic effect in SLE.

Based on the important role anti-dsDNA antibodies play in SLE pathogenesis, approaches to reduce pathogenicity by decreasing or blocking these antibodies may effectively alleviate disease. Current traditional therapies, however, are mainly based on the application of non-specific immunosuppressive drugs, including cytotoxic immunosuppressive agents and corticosteroids, which have extensive clinical use but may have unavoidable serious adverse effects. Based on our improved understanding of the pathogenesis of anti-dsDNA antibodies, attempts have been made to develop promising biologically targeted therapies, like B cell targeted biologics and mimic peptides. In this review, we focus on existing SLE treatment strategies targeting the different pathogenic aspects of anti-dsDNA antibodies, including reducing production and blocking the binding of anti-dsDNA antibodies to antigens (Table 1).

**Table 1:** The effects on anti-dsDNA antibody of different SLE treatments

**Effect on Anti-dsDNA Antibody**	**Treatment**	**Agent**	**Biological Effect**
Reduction	Immunosuppression	Corticosteroids, cyclophosphamide, azathioprine, methotrexate, tacrolimus, mycophenolate mofetil	Inhibition of B cell proliferation
	Immunomodulation	Hydroxychloroquine	Inhibition of auto-nuclear antigen presentation
	Immunoadsorption	Immobilized protein A, DNA-collodion-charcoal membranes, phenylalanine ligand, etc	Removal of pathogenic substances in blood
	CD20 target	Rituximab, ocrelizumab, obinutuzumab	B cell depletion
	CD19 target	Obexelimab	B cell depletion
	CD22 target	Epratuzumab	B cell inactivation and B cell depletion (modest)
	BAFF target	Belimumab, blisibimod, tabalumab	B cell depletion (modest)
	BAFF/APRIL target	Atacicept, telitacicept	B cell and plasma cell depletion
	BTK target	Ibrutinib, fenebrutinib/GDC-0853	Blockade of B cell maturation
	iDC vaccine	DNA antigen-pulsed iDCs	Induction of immune tolerance
Blockade	Mimic peptides	hCDR1, pCons, DWEYS, FISLE-412, ALW	Blockade of anti-dsDNA antibodies binding to antigens and tissues

## The Origin of Anti-dsDNA Antibodies

Nuclear substances originate from billions of dead cells daily due to senescence, infections, or mechanical injuries. Normally, the immune system is not accessible to nuclear antigens because dead cells are quickly cleared to achieve self-stability, avoiding the accumulation of nuclear antigens. In addition, dysregulation of various cell death processes (e.g., apoptosis, necrosis, NETosis, and autophagy) accounts for the exposure of nuclear autoantigens [8]. Dysregulation of dead cells and clearance deficiency have been suggested in SLE patients [1]. Anti-dsDNA antibodies can recognize released dsDNA and compose ICs. Circulating ICs containing nucleic acids stimulate immature dendritic cell (iDC) activation by FcγRs. The activated DCs migrate to the lymphatic tissue where iDCs mature through overexpression of costimulatory molecules (CD80, CD86, and PD-L1), redistribution of MHC class II molecules, and secretion of proinflammatory cytokines (IL‐1β, IL‐6, and IL‐10), which promote activation of B cells and T cells [5, 9]. The differentially expressed FcγRs DCs may also promote the immune response to autoantigens in SLE [10]. Simultaneously, the DNA‐containing antigens can also directly activate autoreactive B cells by both B cell receptors (BCR) and Toll-like receptors (TLRs). In SLE patients, TLRs, especially TLR7 and TLR9, are crucial in the loss of B cell tolerance. They recognize BCR-mediated internalized self-nucleic acids, such as unmethylated CpG motifs in DNA (CpG-DNA) and RNA-associated antigens [11]. The intrinsic TLR7 signaling pathway participates in B cell differentiation via activating autoreactive B cells and promoting the proliferation and differentiation into antibody-producing plasma cells [11, 12]. Dysregulation of innate immunity, particularly over secretion of IFN-α, plays a vital role in somatic mutation and the class switch of anti-dsDNA antibodies in SLE [13]. Thus, plasmacytoid dendritic cells, as efficient producers of IFN-α, are critical in SLE.

Moreover, the autophagy mechanism is associated with SLE pathogenesis. The LC3-associated phagocytosis and beclin-1 autophagy pathways regulate the production of IFN-α [14, 15]. The discrepancy of the anti-dsDNA antibody subclass (IgM, IgG1, IgG2a, IgG2b, and IgG3) has a remarkably different affinity and pathogenicity in SLE [16]. Particular subclasses of anti-DNA antibodies, such as IgG2a, IgG2b, and IgG3, are more closely associated with a kidney’s pathogenic potential and active nephritis [16]. This magnifies the immune response and mediates SLE pathogenesis.

## The Anti-DNA Antibody-Targeting Organs

The anti-dsDNA autoantibody is the hallmark of lupus nephritis. Nearly 80% of lupus nephritis patients have this seropositive trait. Moreover, the effect of anti-dsDNA antibodies on renal resident cells in the lupus nephritis process is clear. Apart from reacting with diverse DNA substances, anti-dsDNA antibodies can bind to variant non-DNA antigens, such as annexin II, α-actinin, laminin, collagen III, collagen IV, entactin, complement receptor type 1 (C1q), N-methyl-D-aspartate receptor (NMDAR), ribosomal P proteins, heparan sulfate, and others [5]. Additionally, SLE patients have anti-dsDNA IgG capable of penetrating cells by binding to cell surface antigens, thus internalizing the autoantibody into the cytoplasm and nucleus [6]. This augments anti-dsDNA IgG to a more prominent pathogenetic role in lupus nephritis.

Anti-dsDNA antibodies contribute to inflammatory and fibrosis processes by overexpressing a wealth of proinflammatory cytokines, such as monocyte chemotactic protein 1 (MCP-1), TNF-α, IL-1β, IL-6, IL-8, hyaluronan, and lipocalin-2. This has been demonstrated in both human and murine mesangial cells and in human proximal tubular epithelial cells (PTECs). This results in a chronic profile of kidney damage [5]. The accumulation of inflammatory cytokines mediated by the anti-dsDNA antibody can also trigger infiltration of immune cells and enhance endoplasmic-reticulum stress in mesangial cells [17].

Multiple signaling pathways are also associated with the development and progression of lupus nephritis. Tumor necrosis factor-like weak inducer of apoptosis (TWEAK) acts through its receptor fibroblast growth factor-inducible 14 (Fn14) to induce downstream inflammatory and fibrotic responses in kidney cells. This includes the expression of MCP-1 and interferon γ-induced protein 10 (IP-10) [18–20]. Inhibition of TWEAK relieves inflammation and protects the filtration barrier by decreasing renal IgG deposition without influencing serum anti-DNA IgG levels [21]. Fibrosis progression is a common manifestation in chronic lupus nephritis, in which the irreversible injury is induced by TWEAK/Fn14 [5]. This process can lead to lupus nephritis proliferation, apoptosis, or fibrosis via different mechanisms.

Although anti-dsDNA antibodies alone are inadequate to cause nephritis, studies show that severe combined immunodeficiency (SCID) mice only manifested proteinuria without pathologic changes to kidney histology after an injection of human anti-DNA IgG antibodies. This confirms that lupus nephritis initiation is not due to a single factor [22]. Nonetheless, a large amount of research suggests that anti-dsDNA antibodies accelerate lupus nephritis processes, and lupus nephritis symptoms are improved by blocking anti-dsDNA antibodies [23, 24]. We conclude that anti-dsDNA antibodies are involved in renal inflammation and fibrosis; however, further investigation is needed on the complex mechanism of the cytokine networks and signaling pathways.

In addition to the kidneys, the central nervous system can be affected by SLE, and neuropsychiatric manifestations are related to a poor prognosis in SLE patients. Neuropsychiatric complications occur in the majority of patients with SLE and may present frequently during SLE onset [25]. Anti-phospholipid, anti-NMDAR, anti-microtubule-associated protein 2, anti-ribosomal P protein, anti-aquaporin 4, anti-endothelial cell, and anti-suprabasin autoantibodies account for central nervous system disease progression [26]. Anti-dsDNA antibodies bind to cross-reactive antigens, NR2A and NR2B subunits of NMDAR, which leads to neuronal cell excitotoxicity and death by increasing the neuronal calcium influx [27]. Disruption of the blood–brain barrier (BBB) might enable anti-dsDNA/anti-NMDAR antibodies in the serum of patients with SLE to access to the central nervous system, contributing to neuronal death and cognitive dysfunction [28]. Although an increased serum level of anti-dsDNA/anti-NMDAR antibodies is not always correlated with neurological dysfunctions or neuropsychiatric activity, a higher titer of anti-dsDNA/anti-NMDAR antibodies is observed in SLE patients with active diffuse neuropsychiatric complications compared to patients with focal neuropsychiatric or non-inflammatory central nervous system disease [26]. In addition to the difference between specific neuropsychiatric disease conditions, this inconsistency may be due to sample discrepancies and the different cognitive evaluation methods applied in various studies.

**Fig. 1:**
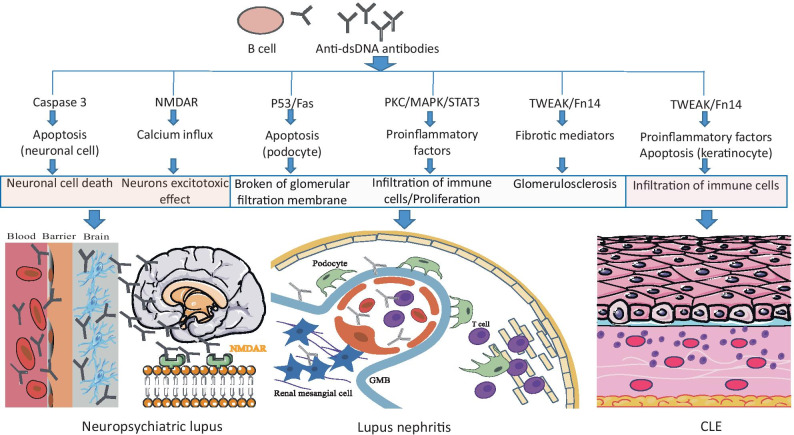
The pathogenic mechanism of anti-dsDNA antibodies in neuropsychiatric lupus, lupus nephritis, and CLE. Anti-dsDNA antibodies can cause the apoptotic cell death of primary neurons by activation of caspase 3 and react with NMDAR, leading to a calcium influx and neuronal excitotoxicity. After binding to DNA and non-DNA antigens, the anti-dsDNA antibodies induce apoptosis via upregulation of the p53 or Fas gene. The internalized anti-dsDNA antibodies activate the protein kinase C (PKC), mitogen-activated protein kinase (MAPK), and signal transducer and activator of transcription (STAT3) signaling pathways; attract immune cells; and enhance cellular proliferation. The deposition of anti-dsDNA IgG, proinflammatory or profibrogenic cytokines, the TWEAK/Fn14, and epithelial mesenchymal transitions (EMT) signaling pathways initiate renal fibrosis in lupus nephritis. Anti-DNA IgG specifically binds to keratinocytes, which promotes the secretion of proinflammatory factors and keratinocyte apoptosis via the TWEAK/Fn14 signaling pathway. This subsequently induces an infiltration of immune cells

Skin involvement is the most common manifestation of SLE, which has a broad spectrum of lesions. Serum anti-dsDNA antibody positivity is observed even in subtypes of patients with cutaneous lupus erythematosus (CLE). It is known that anti-DNA antibodies can specifically bind to the dermal-epidermal junction of the skin and keratinocytes, leading to keratinocyte apoptosis by antibody-dependent cell-mediated cytotoxicity (ADCC) [5]. Anti-DNA IgG in combination with inflammatory reactivity promotes binding to cross-reactive antigens, including collagen III and collagen IV in keratinocytes. The suppression of cytokine signaling (SOCS)-1 and 8 are associated with autoantibody accumulation in the skin [29, 30]. Moreover, activation of the TWEAK/Fn14 signal is prominent in the lesions of CLE patients and murine lupus models [31, 32]. UVB irradiation significantly exaggerates both the binding of anti-DNA IgG to and the expression of Fn14 on keratinocytes, which can interact with TWEAK to upregulate the secretion of proinflammatory factors and promote apoptosis [29, 31]. These upregulated factors, including RANTES (regulated upon activation and normal T cell expression, and secretion), IL-6, IL-8, IP-10, and MCP-1, subsequently amplify the inflammatory reaction by T cells and macrophage infiltration [31, 32]. Anti-DNA IgG is closely associated with CLE, but further studies concerning the specific mechanism are needed. The pathogenic mechanism of anti-dsDNA antibodies in neuropsychiatric lupus, lupus nephritis, and CLE is showed in Fig. 1.

## The Reduction of Anti-dsDNA Antibodies

### Immunosuppressive and Immunomodulatory Therapies

Conventional SLE therapies are based on the use of corticosteroids, which have both anti-inflammatory and immunosuppressive effects. In particular, corticosteroids are effective for active lupus nephritis and other lupus-related manifestations. However, their transient curative effect and undesirable adverse side effects limit their usage [33]. Other cytotoxic immunosuppressive agents, such as cyclophosphamide, azathioprine, methotrexate, tacrolimus, or mycophenolate mofetil single, or a combination with corticosteroids, are utilized extensively in patients with various manifestations of SLE for long-term disease control and to minimize steroid requirements. These agents also have significant limitations and serious adverse events, including cytopenia, infections, and possible malignancy [34].

Hydroxychloroquine, as an immunomodulator, can modulate the immune response without increasing the risk of infection or malignancy. Hydroxychloroquine alone or in combination with steroids and immunosuppressive drugs has been widely used in SLE management to improve patients’ long-term survival by controlling lupus flares and accrual of organ damage. According to the 2019 updated European League Against Rheumatism (EULAR) guidelines for the SLE management, hydroxychloroquine is recommended in all lupus patients unless contraindicated (level of evidence 1b, grade of recommendation A) [35]. Hydroxychloroquine exerts immunomodulatory effect by inhibiting BCR and TLR signaling and the secretion of cytokines to prevent B cell survival and autoantibody production [36–38]. It can also prevent MHC class II-mediated auto-nuclear antigen presentation by inhibiting lysosomal activity [39, 40]. Moreover, hydroxychloroquine exerts an anti-IFN-α effect by interfering with nucleic acid sensor cyclic GMP-AMP synthase (cGAS) to inhibit stimulator of interferon genes (STING) pathway [40, 41]. A prospective cohort study has revealed that hydroxychloroquine can ameliorate classical SLE manifestations, such as skin problems and joint pain by anti-inflammation and decreasing autoantibodies level [42].

### Immunoadsorption Therapies

It is well recognized that SLE has a predominant association with aberrant humoral immunity rather than cellular immunity, which provides the rationale for the application of extracorporeal treatment, such as plasma exchange or immunoadsorption, to remove pathogenic components such as autoantibodies, ICs, and cytokines from the patients’ blood. Extracorporeal therapy has been used for SLE management since the 1970s. Traditional plasma exchange technologies are related to a wider range of component removal compared to immunoadsorption and their use is limited due to nonselective removal patterns and restricted clinical efficacy. The current optimization technique, immunoadsorption plasmapheresis, has a higher selective removal that provides an alternative SLE treatment approach. A variety of pioneering materials for SLE treatment have been developed, including immobilized protein A, phenylalanine ligand, DNA-collodion-charcoal membranes, DNA-immobilized adsorbents, DNA-immobilized nanocellulose-based immune adsorbent, and dextran sulfate ligand, which can clear anti-dsDNA antibodies effectively [43, 44]. Despite the possible side effects of plasma product replacement and catheter-related complications, such as bleeding or catheter-related infection, immunoadsorption still seems to be a safe and beneficial technique in refractory SLE patients, especially in pregnant or lactating patients [44, 45].

### B Cell-Targeting Therapies

B cells play an indispensable role in SLE, not only by generating multiple autoantibodies including anti-dsDNA and being a rich source of cytokines, but also by T cell activation and APC functioning. Based on this fact, therapeutic strategies targeting B cells and reducing their activity are prominent candidates for decreasing anti-dsDNA antibodies and treating SLE.

#### B Cell Surface Antigens

B cells arise from the bone marrow and undergo several stages of maturation until they develop into plasma cells. During maturation, B cells express various cell surface antigens which are useful markers and potential therapy targets [46]. CD20, CD22, and CD19 are B cell surface antigens expressed on immature and mature B cells that are absent from terminally differentiated plasma cells. Upon binding to their target, monoclonal antibodies (mAbs) targeting these B cell surface antigens can lead to B cell apoptosis and depletion, thus decreasing autoantibodies levels.

Rituximab, a chimeric (mouse-human) mAb against CD20, has demonstrated a significant effect on rheumatoid arthritis and B cell malignancies, but its therapeutic effects on SLE remain controversial [47–49]. The effectiveness of rituximab in the treatment of refractory lupus nephritis and severe non-renal SLE manifestations (including severe joint, hematological, cutaneous, and neuropsychiatric disease) has been demonstrated in many observational studies and routine clinical uses [50–53].

However, two large, phase III, randomized placebo-controlled trials of rituximab in extra-renal lupus (EXPLORER study, NCT00137969) and renal lupus (LUNAR study, NCT00282347) failed to meet their primary endpoints [54, 55]. LUNAR study, including 144 patients with class III or class IV lupus nephritis, is the largest randomized, placebo-controlled study to evaluate the effect of rituximab to initial therapy for proliferative lupus nephritis [55]. LUNAR revealed that the overall (complete and partial) renal response rates were 45.8% among the 72 patients receiving placebo and 56.9% among the 72 patients receiving rituximab; but this study did not demonstrate a statistically significant difference between the responses of patients treated with rituximab and those of patients treated with placebo after 1 year of treatment [55]. In contrast to the effect of rituximab in terms of the clinical endpoints, rituximab significantly improved anti-dsDNA antibody and serum complement C3/C4 levels in LUNAR study [55].

These contradictory results of rituximab are somewhat unexpected; factors surrounding clinical trial design and size, background medications, and the complexity and heterogeneity of SLE make it difficult to determine its true efficacy and may explain the discrepancies in different trials [49]. Nevertheless, based on the fact that sufficient clinical data demonstrates the effectiveness and safety of rituximab in difficult-to-treat lupus patients, both the American College of Rheumatology (ACR) and the EULAR recommend rituximab as an appropriate option for refractory SLE patients with lupus nephritis or hematological damage, especially after conventional therapy has failed [56–59].

Two fully humanized anti-CD20 mAbs have also been studied in SLE to avoid high rate of allergy-like responses to rituximab in patients with SLE [49]. Ocrelizumab is a recombinant humanized mAb that selectively targets and depletes CD20 + B cells in the peripheral circulation and has been studied in two clinical trials in SLE [49, 60]. A phase III trial in non-renal SLE (BEGIN study) was terminated early when the sponsor decided not to pursue this indication [49]. Another randomized, double-blind, phase III trial (BELONG study, NCT00626197) evaluates ocrelizumab in SLE patients with lupus nephritis who were receiving ocrelizumab and either cyclophosphamide or mycophenolate mofetil [61]. The 32-week data revealed renal response rates of 63% and 51% in the ocrelizumab and placebo groups respectively, and an apparent benefit in patients receiving the background cyclophosphamide [61]. However, due to a high rate of serious infection in patients receiving background mycophenolate mofetil, BELONG study terminated early [61]. Obinutuzumab, another fully humanized anti-CD20 mAb, is more efficient than rituximab at inducing B cell cytotoxicity in patients with rheumatoid arthritis (RA) or SLE, and has just completed its phase II trial (NCT02550652) [62].

Obexelimab (XmAb5871), a novel humanized anti-CD19 mAb, has been evaluated the ability to maintain disease activity in 105 patients with moderate-to-severe SLE (without organ threatening) in a phase II trial (NCT02725515). An assessment of the data from this trial revealed that patients treated with obexelimab have a better control of disease activity level compared to patients treated with placebo [49]. An anti-CD22 mAb called epratuzumab modulates B cell signaling without substantial reductions in the number of B cells. Two large, phase III, randomized placebo-controlled trials in patients with moderate-to-severe active SLE (EMBODY 1, NCT01262365 and EMBODY 2 study, NCT01261793) revealed that treatment with epratuzumab did not result in improvements in the rate of positive response over that observed in the placebo group [63]. It is not clear why trials have failed to demonstrate efficacy of epratuzumab, but further evaluation of patient subsets, biomarkers, and exploratory outcome models may improve the design of future SLE clinical trials and test the true level of effectiveness.

#### BAFF/APRIL

There are two B-lineage cell survival factors, B cell activating factor (BAFF) and a proliferation-inducing ligand (APRIL). They involved in B cell maturation, activation, and survival and have received the bulk of attention in SLE therapy [64]. BAFF, also known as B lymphocyte stimulator (BLyS), is a 285-amino acid transmembrane protein member of the tumor necrosis factor ligand superfamily [65]. The soluble form BAFF can bind to three receptors on the surface of B cells: B cell maturation antigen (BCMA), transmembrane activator and cyclophilin interactor (TACI), and BAFF receptor 3 (BR3) [65]. BAFF leads to a greater dependency of autoreactive B cells than of normal non-autoreactive B cells [66]. Genetic depletion or BAFF antagonist treatment reduces B cells and prevents progression of SLE in MRL/lpr or BWF1 mice [67]. In addition, high sera BAFF levels are common in SLE and closely associated with higher disease activity or autoantibody levels in SLE patients [68]. Moreover, patients with BAFF overexpression have a greater tendency towards organ damage over time compared to patients with lower BAFF expression levels [69]. APRIL, another B-lineage cell survival factor, is a 250-amino acid with similar biological properties to BAFF that binds to TACI and BCMA, but not to BR3 [70]. While BAFF plays a profound role in SLE, the cogent evidence in lupus-prone murine and SLE patients shows this is not the case with APRIL. Although APRIL is an essential survival factor with substantial homology to BAFF, neither APRIL-transgenic or APRIL-deficient NZM mice demonstrated an appreciated effect on B cell numbers and clinical autoimmune features [71]. Combination treatment of BAFF and APRIL antagonists is not more effective compared to BAFF antagonist treatment alone in NZM mice [72, 73]. Based on the comprehensive data, APRIL may not be as vital as BAFF in SLE.

Therapies were developed based on BAFF’s general properties. Belimumab, a fully humanized recombinant IgG1 mAb, binds and antagonizes to soluble BAFF, blocking its interaction with BCMA, TACI, and BR3, therefore inhibiting BAFF activity [64]. Two large placebo-controlled phase III trials, BLISS-52 and BLISS-76, studied the efficacy in patients with mild-to-moderate SLE (without severe active lupus nephritis and central nervous system manifestation) and demonstrated a significant amelioration of disease activity with 10 mg/kg of intravenous belimumab as compared with placebo. Moreover, belimumab also met key secondary endpoints, particularly reducing the time to severe flares, corticosteroid-sparing effect, fatigue levels, and improvements in health-related quality of life (HRQoL) [69, 74]. Belimumab, based on its significant clinical response and safety in several trails, was FDA-approved for SLE in March of 2011 [75]. Additionally, a recent retrospective study also showed that patients with active SLE and low baseline damage can benefit from belimumab early in the disease [76].

In a randomized, controlled trial (BLISS-LN) including 448 adult patients with active lupus nephritis, belimumab plus standard therapy compared to standard therapy (mycophenolate mofetil or cyclophosphamide-azathioprine) alone had a higher primary efficacy renal response [77]. However, the beneficial effect in patients with severe active central nervous system disease and the potential side effects, such as the rate of infections originated from B cell depletion, still require additional confirmation in further trials [69]. The combination treatment studies of B cell depletion with rituximab and B cell survival inhibition with belimumab demonstrated a better clinical effect [75]. However, given the conflicting evidence, the usefulness of combination therapy must be examined in the future.

Four other BAFF targeting biological agents are in development. Blisibimod, a Fc fusion protein of BAFF binding domains, binds to both soluble and membrane-bound BAFF [78]. Tabalumab is a human IgG4 mAb that binds to both forms of BAFF and is provided subcutaneously [79]. Atacicept, a fully humanized soluble fusion protein containing the Fc portion of IgG and the TACI receptor, binds to both forms of BAFF and APRIL [80]. The fourth BAFF antagonist, telitacicept (RC18), a recombinant fusion protein of the human IgG1 Fc domain and TACI receptor extracellular domain that binds to BAFF, is in the phase II trial recruitment phase in the treatment of IgA nephropathy (NCT04905212).

#### BTK

Bruton’s tyrosine kinase (BTK) is a tyrosine-protein kinase expressed in B cells and myeloid cells. Functionally, BTK is an essential molecule involved in both innate and adaptive immunity. It regulates many signal transduction pathways, such as the B cell receptor and Fcγ receptor signal [81]. As a critical molecule for regulating B cell differentiation and activation, genetic defects of BTK contribute to X-linked agammaglobulinemia, a severe disorder involving the absence of mature B cells and immunoglobulins in human [81]. In multiple preclinical studies of murine lupus, several distinct BTK inhibitors (including ibrutinib, which has been widely used in the treatment of several B cell malignancies, and fenebrutinib/GDC-0853) reduced splenic germinal center B cells, plasma cells, and various autoantibodies levels, including anti-dsDNA autoantibodies [49, 82]. Correspondingly, the overexpression of BTK led to an increased number of B cells and anti-nuclear autoantibodies, manifested as a series of SLE-like damage [83]. Whether BTK inhibitors can improve SLE manifestation in humans must be confirmed, but they are still a promising strategy.

#### iDC Vaccine

The deficiency of immune tolerance is key in SLE pathogenesis. Therefore, iDCs that induce the clonal anergy of T cells have been highlighted. DCs are highly specialized APCs which present MHC-antigen complex as the first active signal and provide costimulatory factors as the second signal to T cells for clone activity [84]. Whether iDCs can induce apoptosis of T cells and differentiate T regulatory cells (Treg) depends on the levels of MHC molecules, CD80, and CD86 [85]. In short, the immune response induced by DCs depends on the mature state. Based on its immunomodulatory properties, a strategy has been developed for SLE by iDCs loaded with specific antigen-including dsDNA as a live cell vaccine [86]. In a preclinical study, DNA antigen-pulsed iDC vaccine demonstrated a protective effect on renal damage by significantly decreasing proteinuria, blood urea nitrogen (BUN), serum creatinine (SCr), and renal antibody deposition. However, the exact mechanism of iDC vaccine interacting with immunity is unclear; iDC vaccine still requires further investigation.

## Blockade of Anti-dsDNA Antibodies

Based on the pathogenic mechanism of anti-dsDNA antibodies and the risk of infection using B cell depleting agents, therapeutic small-molecule mimics of peptides that target anti-dsDNA antibodies and block their interaction with antigens or tissues are getting attention. Synthetic peptides are selected based on the specific sequences of pathogenic antibody. The desired amino acid residues can be selected to ensure a higher affinity and lower immunogenicity [5]. Below, we discuss mimic peptides that block the binding of anti-dsDNA antibodies and inhibit SLE-associated autoimmune reactions (Table 2).

**Table 2:** Characteristics and effects of mimic peptides for SLE treatment

**Peptide**	**Origin**	**Molecular Mechanism**	**In Vivo Effects**	**Administration Route**	**Clinical trial**
hCDR1 (Edratide)	Synthetic murine anti-dsDNA mAb	↓ IL-1β, IFN-γ, IFN-α, TNF-α, IL-10, BAFF, caspase3, caspase8 ↑ TGF-β, SOCS-1 ↓ T cell apoptosis ↓ T cell activation ↑ CD4 Tregs, CD8 Tregs ↑ B cell apoptosis ↓ B cell activation Induction of tolerogenic dendritic cells	↓ Anti-dsDNA antibodies ↓ Anti-nuclear antibodies ↓ Anti-cardiolipin antibodies Amelioration of renal and central nervous system manifestations Prolonged survival	Subcutaneous route	Double-blind, Phase II, placebo-controlled clinical trial
pCons	Based on CDR1 of a human anti-DNA mAb	↓ IFN-γ, IL-4 ↑ CD4 Tregs, CD8 Tregs	↓ Anti-dsDNA antibodies ↓ Anti-nucleosome antibodies ↓ Anti-cardiolipin antibodies Delayed onset of nephritis Prolonged survival	Intravenous route	N/A
DWEYS	Selected by phage library with murine anti-dsDNA mAb (D/E W D/E Y S/G shares consensus sequence with NMDAR)	↓ Binding of anti-dsDNA antibodies to dsDNA, DWEYS ↓ Glomerular deposition of anti-dsDNA antibodies Inhibition of autoreactive B cells	↓ Anti-dsDNA antibodies Amelioration of renal and central nervous system manifestations	Intravenous route	N/A
FISLE-412	Molecular topology of DWEYS	↓ Binding of anti-dsDNA antibodies to dsDNA, DWEYS, cardiolipin ↓ Glomerular deposition of anti-dsDNA antibodies ↓ Neuronal apoptosis	↓ Anti-dsDNA antibodies Amelioration of renal and central nervous system manifestations	Oral route	N/A
ALW	Selected by phage library with four types of murine anti-dsDNA IgG mAbs	↓ TGF-β, PDGF-B, CTGF ↓ Binding of anti-dsDNA antibodies to dsDNA, laminin ↓ Glomerular deposition of anti-dsDNA antibodies ↓ Infiltration of inflammatory cells in renal tissue	↓ Anti-dsDNA antibodies Amelioration of renal manifestations	Intravenous route	N/A

*SOCS-1* suppression of cytokine signaling-1, *PDGF-B* platelet-derived growth factor-B, *CTGF* connective tissue growth factor, *N/A* not available

### hCDR1

hCDR1 (GYYWSWIRQPPGKGEEWIG) is a 19-mer peptide that is based on the heavy chain complementarity-determining region 1 (CDR1) sequences from human monoclonal anti-DNA antibody [87]. The potential mechanism of its therapeutic effect has been well documented. hCDR1 exerts protective effects by regulating various cytokines and molecules, including downregulating proinflammatory cytokines such as IL-1β, IFN-α, IFN-γ, IL-10, and TNF-α and upregulating immunosuppressive cytokine transforming growth factor-β (TGF-β). LFA-1 and CD44, expressed on APC for T cell interactions, are also downregulated [75]. hCDR1 induces peripheral tolerance, which is involved with various immune cells, including regulating Tregs and inducing DC with an immature or tolerogenic phenotype [75, 88]. c-Jun NH2-terminal kinase (JNK) which is part of the p21Ras/MAP kinase pathway is highly expressed in the T cells of lupus mice [89]. hCDR1 can significantly decrease the rate of T cell apoptosis by affecting JNK [89]. hCDR1 can also upregulate the anti-apoptotic molecule Bcl-xL and diminish caspase-3 to prevent T cell apoptosis [89]. hCDR1 can reduce anti-dsDNA antibodies by affecting B cell survival and autoreactivity through BAFF inhibition [90]. However, it acts in a more modest way than BAFF inhibitors [90]. A recent study shows that hCDR1 can downregulate the expression of the indoleamine 2, 3-dioxygenase (IDO) gene, which increases activity in SLE patients [91].

Various murine lupus models reveal that weekly subcutaneous administration of hCDR1 at a small dose (25-100 ug) ameliorates lupus manifestations, including reducing serological autoantibodies and relieving renal damage [92, 93]. Meanwhile, treatment with hCDR1 can not only improve brain pathology but also ameliorate cognitive function and mood-related behaviors in lupus-prone mice [94]. In a randomized, placebo-controlled phase II clinical trial, the involved 340 SLE patients (mainly with musculoskeletal, hematological, skin, or mucous membrane manifestations and without active lupus nephritis or central nervous system manifestations) are given a subcutaneous administration of hCDR1/Edratide at 0.5, 1.0, or 1.5 mg or placebo once a week [95]. Although the primary endpoints of the SLE disease activity index (SLEDAI-2 K) were not met, this trial showed that hCDR1/Edratide is safe and well tolerated, and exerts remarkable beneficial effects by secondary British Isles Lupus Assessment Group index (BILAG) score [95]. Therefore, further studies using an appropriate endpoint in SLE patients should be carried out and the effect of hCDR1 in more severe types of SLE needs to be clarified.

### pConsensus

pConsensus (pCons, FIEWNKLRFRQGLEW) is a 15-amino acid peptide that is derived from the heavy chain variable region of murine anti-dsDNA antibody [96]. pCons, acting as a tolerogen in vivo, can delay the onset of nephritis and prolong survival time significantly in lupus-prone mice (NZB/NZW). This effect was associated with the decreased production of autoantibodies and regulation of T cell autoreactivity, such as induction of CD4^+^CD25^+^ Tregs and inhibitory CD8^+^ T cells [96, 97]. While incubated with peripheral blood mononuclear cells (PBMC) in SLE patients, pCons can expand CD4^+^CD25^+^ Tregs and suppress proliferation and secretion of inflammatory cytokines [98]. Moreover, the oral dosage form is not expected to induce immunogenicity [99]. Oral administration with modified pCons consisting of D-amino acids can ameliorate proteinuria and serum anti-dsDNA antibodies in lupus-prone mice in a safe and effective manner [99].

### DWEYS

The DWEYS (or consensus sequence D/E W D/E Y S/G) peptide, selected by the phage library with mouse monoclonal anti-dsDNA R4A mAb, inhibits R4A mAb from binding to dsDNA. Moreover, DWEYS is also part of the NMDAR expressed on neurons, which has demonstrated that anti-DWEYS and anti-dsDNA antibodies have cross-reactivity with NMDAR [100]. Intravenous administration of the DWEYS peptide to gestating mice demonstrates a protective effect on fetal brains when exposed to toxic doses of anti-NMDAR antibodies [101]. Ex vivo human lupus studies indicate that the therapeutic potential of the DWEYS peptide comes from inhibiting DNA binding [101]. DWEYS peptide can reduce anti-dsDNA antibody titers and ameliorate nephritis in lupus-prone mice by suppressing autoreactive B cells [102]. The DWEYS peptide appears to be harmless when administered intravenously, which is likely due to its non-immunogenicity [102]. However, its unstable construction means that it cannot be administered orally and the short half-life compromises its potential effect.

### FISLE-412

FISLE-412, a molecular topology of the DWEYS structure, has similar mimic-neutralizing activities as the DWEYS peptide. FISLE-412 demonstrated its effectiveness in vitro, ex vivo, and in vivo [103, 104]. It can block DNA recognition of anti-dsDNA antibodies, reduce glomerular antibody deposition, and ameliorate neurotoxicity by inhibiting the neuronal apoptosis induced by R4A mAb in the mouse hippocampus with a noticeably superior effect compared to DWEYS [104]. The analogues of FISLE-412 have also been identified to neutralize anti-dsDNA antibodies more efficiently by establishing a mimic epitope library around FISLE-412 [105]. The peptides’ small molecular property has advantages in high-throughput and standardized synthesis technology. A practical, simplified synthetic method has been attempted to achieve rapid and cheap synthesis [106]. FISLE-412 was well tolerated and had no toxicity nor immunogenicity [105]. Its stable structure also makes FISLE-412 suitable for oral delivery, which is another advantage over DWEYS.

### ALW

ALW (ALWPPNLHAWVP), a 12-mer peptide mimic, was selected from the four types of murine monoclonal anti-DNA IgG isotypes (IgG1, IgG2a, IgG2b, and IgG3) by screening the phage display libraries [24]. ALW peptide can bind to all four IgG isotypes with different affinity and prevent IgG isotypes from binding to antigens of dsDNA and laminin. This molecule can also prevent IgG isotypes or anti-dsDNA antibodies in SLE patients’ serum interacting with glomerulus and glomerular mesangial cells [24]. ALW peptides can inhibit glomerular deposition of antibodies, reduce serum anti-dsDNA antibody titers, improve renal pathological manifestations, or suppress proliferation, fibrosis, and inflammatory cell infiltration of renal tissue, providing a protective effect on lupus nephritis manifestation in MRL/lpr mice [23].

Furthermore, alanine substitutions at the third or eighth position decrease binding to anti-dsDNA antibodies [24]. Due to the absence of certain amino acid residues, such as methionine, cysteine, and glutamine, ALW was physiologically stable and resistant to oxidation, cyclization, and degradation. The ALW peptide is non-toxic and non-immunogenic, and has high solubility in water, which makes it safe and convenient to intravenous administration [24]. However, the ALW half-life is only 0.32 h, and the short half-life compromises its efficacy [24]. In future, chemical modifications or nanotechnological delivery carriers may provide a good idea to improve its stability and half-time.

## Conclusions

Anti-dsDNA antibody, the hallmark of SLE, contributes to kidney, brain, and skin damage in SLE. The pathogenicity of autoantibodies had been used to develop a means of attempts to reduce anti-dsDNA antibodies, including immunosuppression, immunoadsorption, B cell-targeting, and iDC vaccine therapies. However, immunosuppression and B cell-targeting therapies are not distinctive for lupus and may hamper normal immune system functioning, which bring unavoidable adverse effects. Notably, the mimic peptides designed by blocking anti-dsDNA antibodies highlight the promising therapeutic potential to ameliorate the manifestation of SLE. The peptides’ small molecular property demonstrates advances in high-throughput and standardized and modified synthesis technology. However, most of the peptides are now at the preclinical stage and have a short half-life and unsatisfactory physiological stability. Further studies are warranted to develop more effective therapies for SLE.

## Data Availability

The datasets used and/or analyzed during the current study are available from the corresponding authors on reasonable request.

## Abbreviations

SLE: Systemic lupus erythematosus
APC: Antigen-presenting cell
IC: Immune complex
iDC: Immature dendritic cell
TLR: Toll-like receptor
NMDAR: N-methyl-D-aspartate receptor
MCP-1: Monocyte chemotactic protein 1
TWEAK: Tumor necrosis factor-like weak inducer of apoptosis
Fn14: Fibroblast growth factor-inducible 14
IP-10: Interferon γ-induced protein 10
CLE: Cutaneous lupus erythematosus
mAb: Monoclonal antibody
BAFF: B cell activating factor
APRIL: A proliferation-inducing ligand
BLyS: B lymphocyte stimulator
BCMA: B cell maturation antigen
TACI: Transmembrane activator and cyclophilin interactor
BR3: BAFF receptor 3
BTK: Bruton’s tyrosine kinase
Treg: T regulatory cell
CDR1: Complementarity-determining region 1
